# Supplementary material for: Analysis of Complete Chloroplast Genome: Structure, Phylogenetic Relationships of Galega orientalis and Evolutionary Inference of Galegeae
Source: Genes (Basel). 2023 Jan 9;14(1):176. doi: 10.3390/genes14010176 (PMC9859028; doi:10.3390/genes14010176)

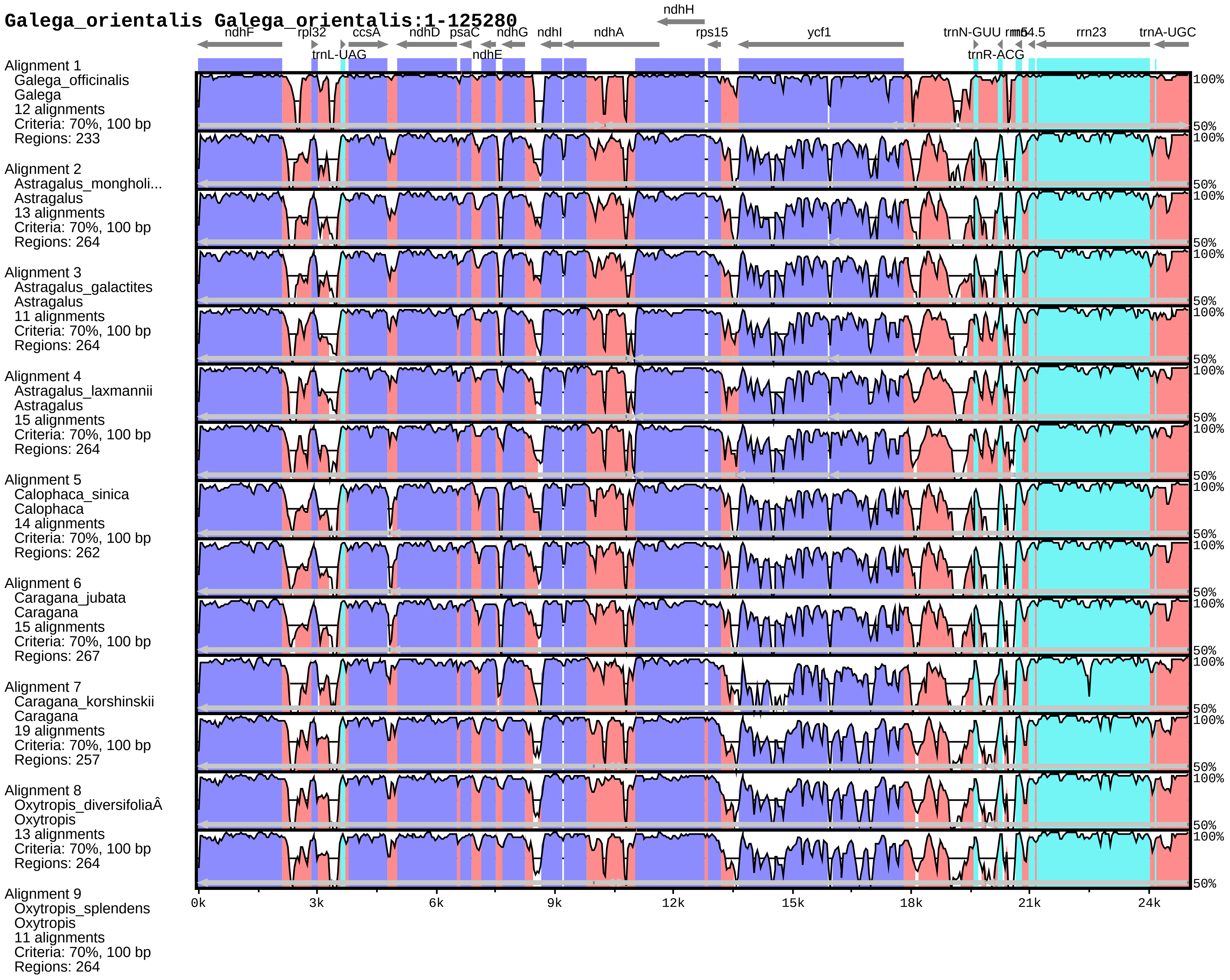
Galega_orientalis Galega_orientalis:1-125280
ndhH
ndhF
rpl32
ccsA
ndhD
psaC
ndhG
ndhI
ndhA
rps15
ycf1
trnN-GUU
rrn5
rrn4.5
rrn23
trnA-UGC
trnL-UAG
ndhE
trnR-ACG
Alignment 1
Galega_officinalis
Galega
12 alignments
Criteria: 70%, 100 bp
Regions: 233
Alignment 2
Astragalus_mongholi...
Astragalus
13 alignments
Criteria: 70%, 100 bp
Regions: 264
Alignment 3
Astragalus_galactites
Astragalus
11 alignments
Criteria: 70%, 100 bp
Regions: 264
Alignment 4
Astragalus_laxmannii
Astragalus
15 alignments
Criteria: 70%, 100 bp
Regions: 264
Alignment 5
Calophaca_sinica
Calophaca
14 alignments
Criteria: 70%, 100 bp
Regions: 262
Alignment 6
Caragana_jubata
Caragana
15 alignments
Criteria: 70%, 100 bp
Regions: 267
Alignment 7
Caragana_korshinskii
Caragana
19 alignments
Criteria: 70%, 100 bp
Regions: 257
Alignment 8
Oxytropis_diversifoliaÂ
Oxytropis
13 alignments
Criteria: 70%, 100 bp
Regions: 264
Alignment 9
Oxytropis_splendens
Oxytropis
11 alignments
Criteria: 70%, 100 bp
Regions: 264
100%
50%
0k
3k
6k
9k
12k
15k
18k
21k
24k

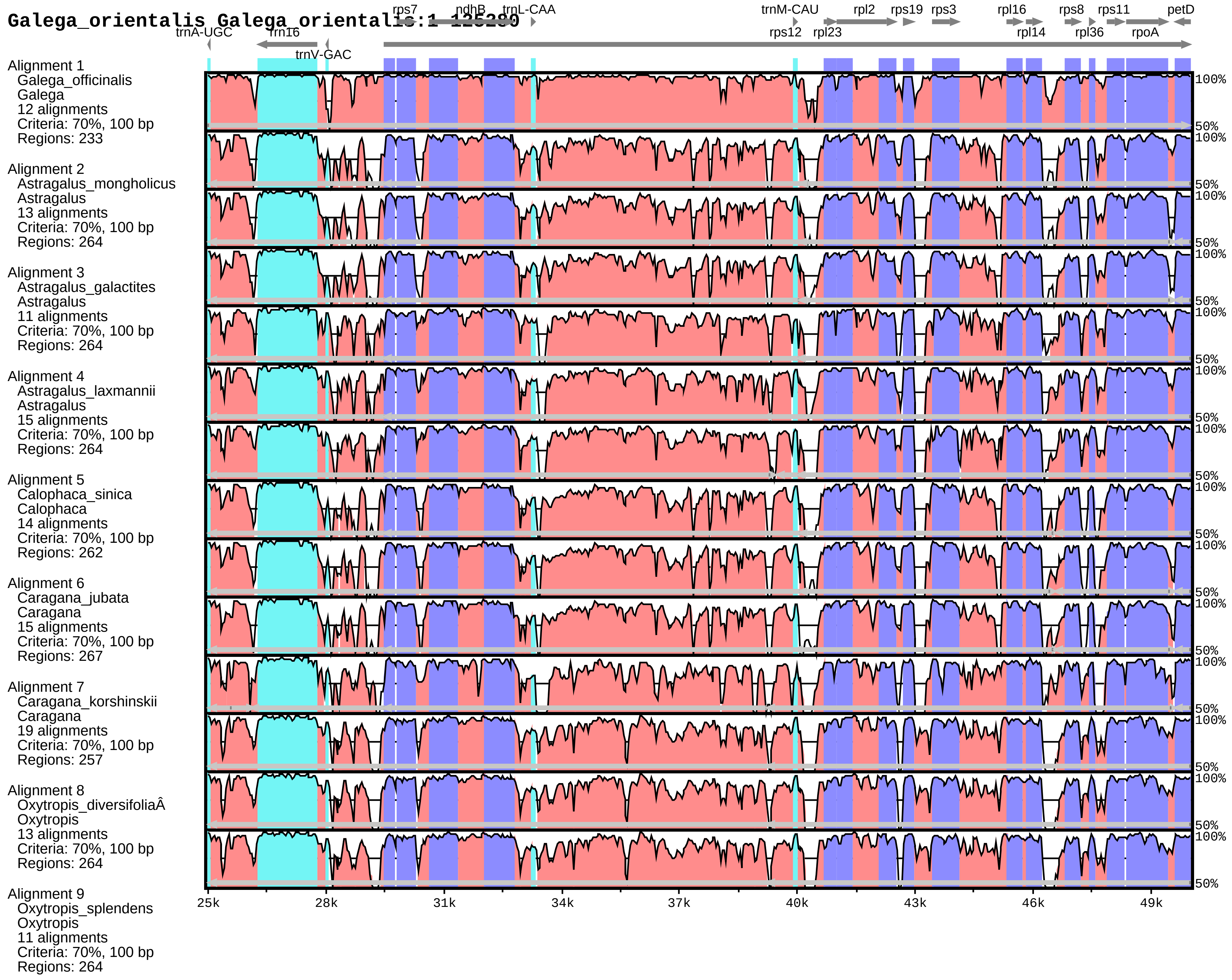

Galega_orientalis Galega_orientalis:1-125280
trnA-UGC
rrn16
trnV-GAC
rps7
ndhB
trnL-CAA
trnM-CAU
rps12
rpl23
rpl2
rps19
rps3
rpl16
rpl14
rps8
rpl36
rps11
rpoA
petD
Alignment 1
Galega_officinalis
Galega
12 alignments
Criteria: 70%, 100 bp
Regions: 233
Alignment 2
Astragalus_mongholicus
Astragalus
13 alignments
Criteria: 70%, 100 bp
Regions: 264
Alignment 3
Astragalus_galactites
Astragalus
11 alignments
Criteria: 70%, 100 bp
Regions: 264
Alignment 4
Astragalus_laxmannii
Astragalus
15 alignments
Criteria: 70%, 100 bp
Regions: 264
Alignment 5
Calophaca_sinica
Calophaca
14 alignments
Criteria: 70%, 100 bp
Regions: 262
Alignment 6
Caragana_jubata
Caragana
15 alignments
Criteria: 70%, 100 bp
Regions: 267
Alignment 7
Caragana_korshinskii
Caragana
19 alignments
Criteria: 70%, 100 bp
Regions: 257
Alignment 8
Oxytropis_diversifoliaÂ
Oxytropis
13 alignments
Criteria: 70%, 100 bp
Regions: 264
Alignment 9
Oxytropis_splendens
Oxytropis
11 alignments
Criteria: 70%, 100 bp
Regions: 264
100%
50%
25k
28k
31k
34k
37k
40k
43k
46k
49k

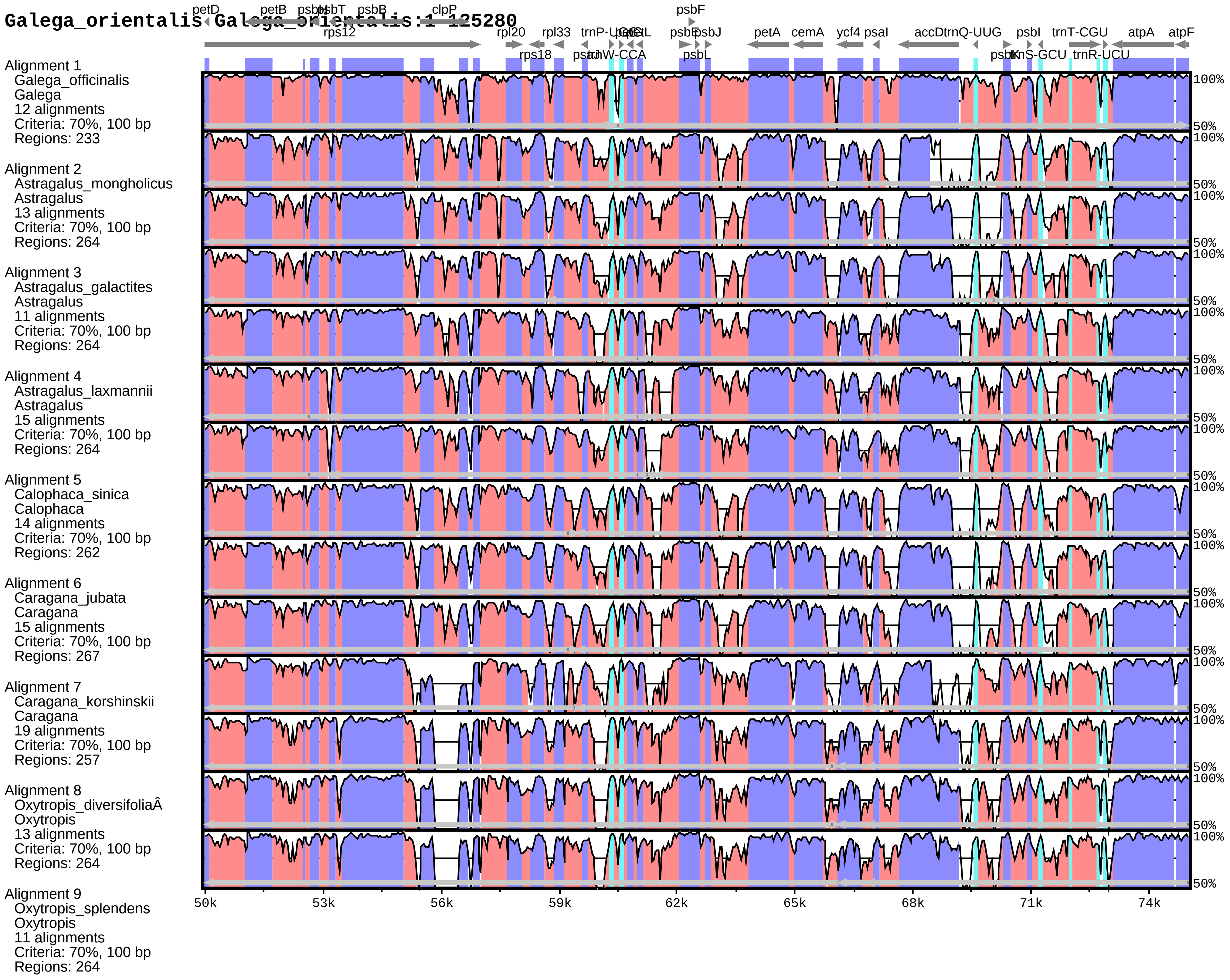
Galega_orientalis Galega_orientalis:1-125280
petD
petB
psbH
psbT
psbB
clpP
rps12
rpl20
rps18
rpl33
psaJ
trnP-UGG
trnW-CCA
petG
petL
psbF
psbE
psbJ
psbL
petA
cemA
ycf4
psaI
accD
trnQ-UUG
psbK
psbI
trnS-GCU
trnT-CGU
trnR-UCU
atpA
atpF
Alignment 1
Galega_officinalis
Galega
12 alignments
Criteria: 70%, 100 bp
Regions: 233
Alignment 2
Astragalus_mongholicus
Astragalus
13 alignments
Criteria: 70%, 100 bp
Regions: 264
Alignment 3
Astragalus_galactites
Astragalus
11 alignments
Criteria: 70%, 100 bp
Regions: 264
Alignment 4
Astragalus_laxmannii
Astragalus
15 alignments
Criteria: 70%, 100 bp
Regions: 264
Alignment 5
Calophaca_sinica
Calophaca
14 alignments
Criteria: 70%, 100 bp
Regions: 262
Alignment 6
Caragana_jubata
Caragana
15 alignments
Criteria: 70%, 100 bp
Regions: 267
Alignment 7
Caragana_korshinskii
Caragana
19 alignments
Criteria: 70%, 100 bp
Regions: 257
Alignment 8
Oxytropis_diversifoliaÂ
Oxytropis
13 alignments
Criteria: 70%, 100 bp
Regions: 264
Alignment 9
Oxytropis_splendens
Oxytropis
11 alignments
Criteria: 70%, 100 bp
Regions: 264
100%
50%
50k
53k
56k
59k
62k
65k
68k
71k
74k

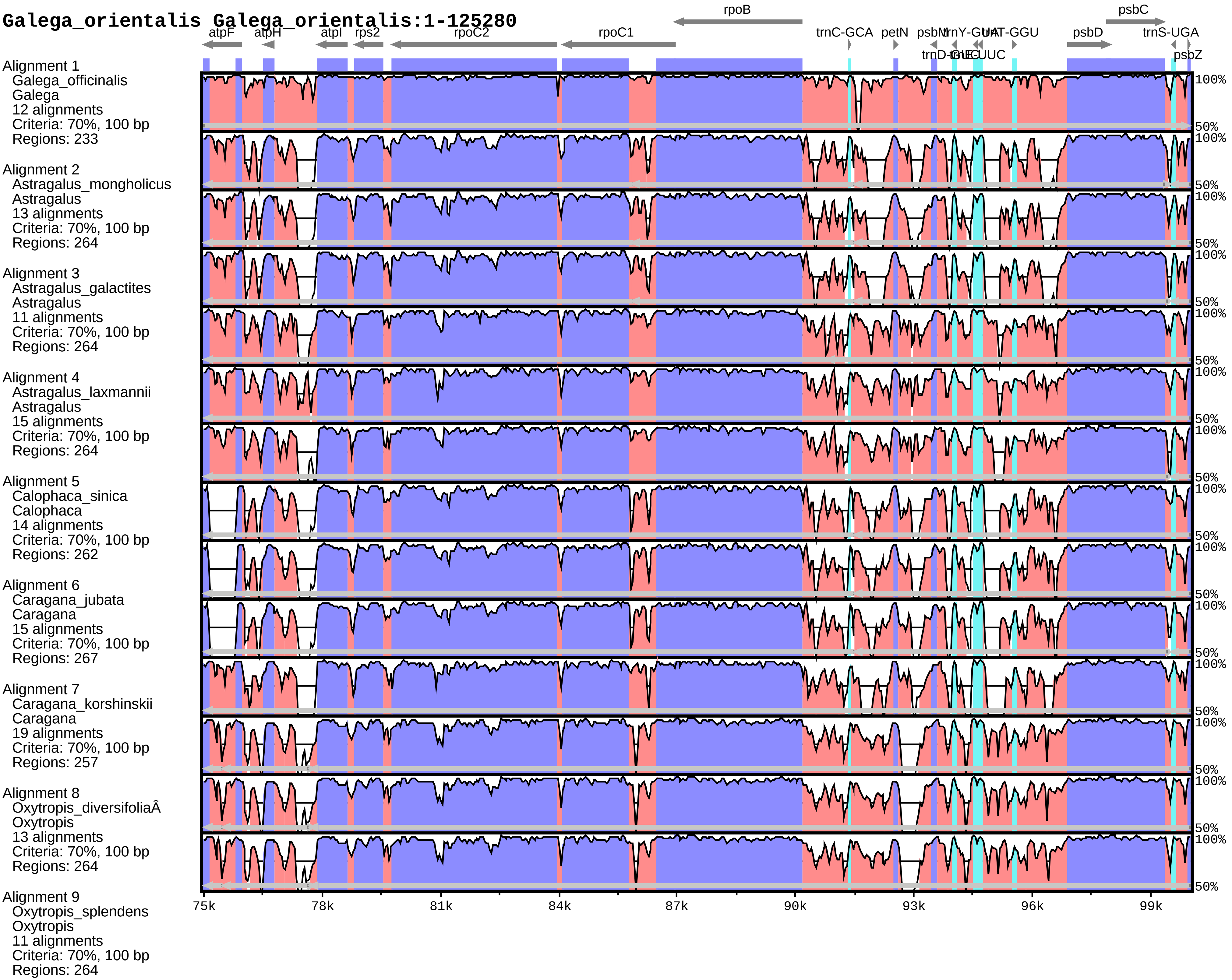

Galega_orientalis Galega_orientalis:1-125280
atpF
atpH
atpI
rps2
rpoC2
rpoC1
rpoB
trnC-GCA
petN
psbM
trnY-GUA
trnT-GGU
trnD-GUC
trnE-UUC
psbD
psbC
trnS-UGA
psbZ
Alignment 1
Galega_officinalis
Galega
12 alignments
Criteria: 70%, 100 bp
Regions: 233
Alignment 2
Astragalus_mongholicus
Astragalus
13 alignments
Criteria: 70%, 100 bp
Regions: 264
Alignment 3
Astragalus_galactites
Astragalus
11 alignments
Criteria: 70%, 100 bp
Regions: 264
Alignment 4
Astragalus_laxmannii
Astragalus
15 alignments
Criteria: 70%, 100 bp
Regions: 264
Alignment 5
Calophaca_sinica
Calophaca
14 alignments
Criteria: 70%, 100 bp
Regions: 262
Alignment 6
Caragana_jubata
Caragana
15 alignments
Criteria: 70%, 100 bp
Regions: 267
Alignment 7
Caragana_korshinskii
Caragana
19 alignments
Criteria: 70%, 100 bp
Regions: 257
Alignment 8
Oxytropis_diversifoliaÂ
Oxytropis
13 alignments
Criteria: 70%, 100 bp
Regions: 264
Alignment 9
Oxytropis_splendens
Oxytropis
11 alignments
Criteria: 70%, 100 bp
Regions: 264
100%
50%
75k
78k
81k
84k
87k
90k
93k
96k
99k

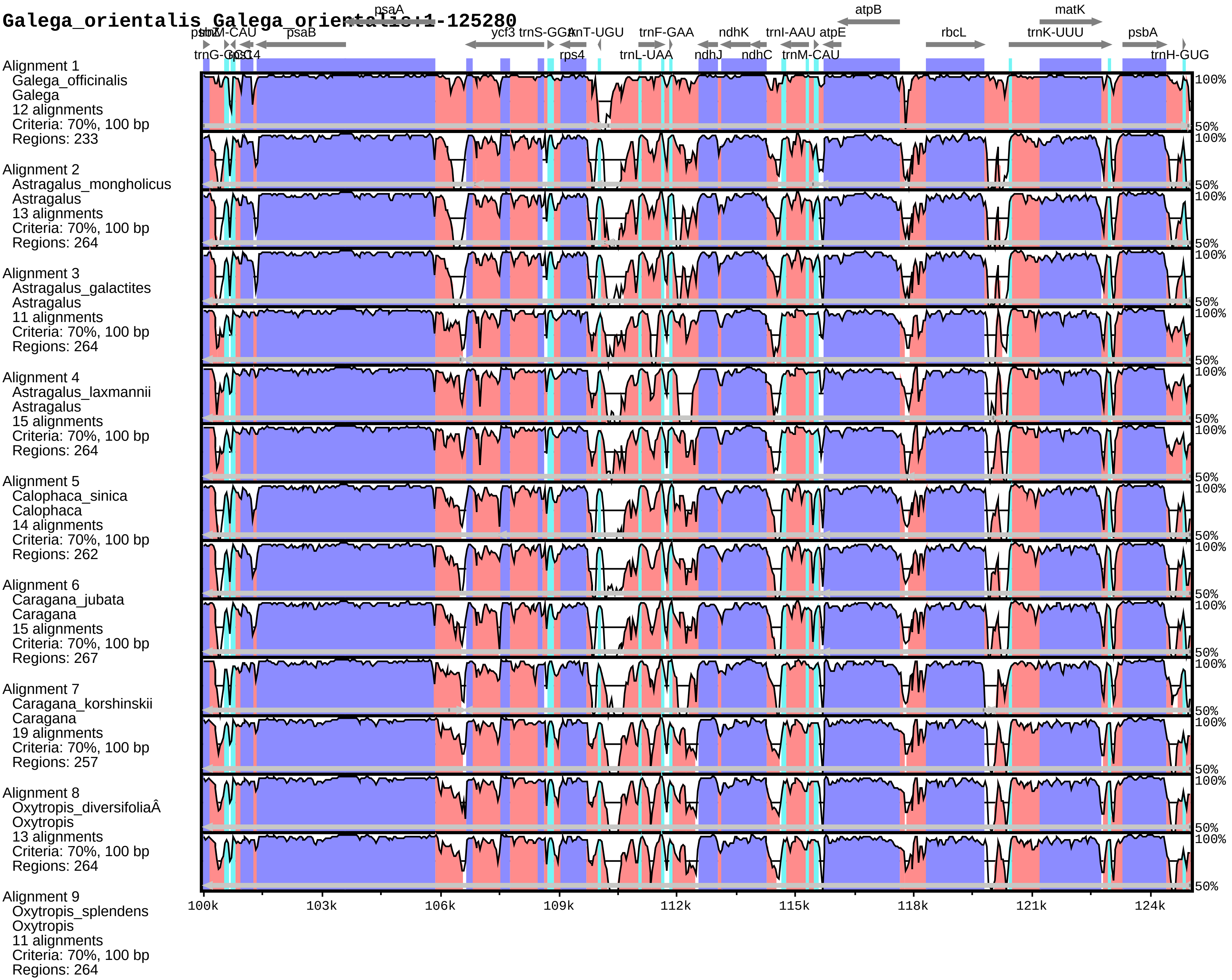
Galega_orientalis Galega_orientalis:1-125280
psaA
atpB
matK
psbZ
trnM-CAU
psaB
ycf3
trnS-GGA
trnT-UGU
trnF-GAA
ndhK
trnI-AAU
atpE
rbcL
trnK-UUU
psbA
trnG-GCC
rps14
rps4
trnL-UAA
ndhJ
ndhC
trnM-CAU
trnH-GUG
Alignment 1
Galega_officinalis
Galega
12 alignments
Criteria: 70%, 100 bp
Regions: 233
Alignment 2
Astragalus_mongholicus
Astragalus
13 alignments
Criteria: 70%, 100 bp
Regions: 264
Alignment 3
Astragalus_galactites
Astragalus
11 alignments
Criteria: 70%, 100 bp
Regions: 264
Alignment 4
Astragalus_laxmannii
Astragalus
15 alignments
Criteria: 70%, 100 bp
Regions: 264
Alignment 5
Calophaca_sinica
Calophaca
14 alignments
Criteria: 70%, 100 bp
Regions: 262
Alignment 6
Caragana_jubata
Caragana
15 alignments
Criteria: 70%, 100 bp
Regions: 267
Alignment 7
Caragana_korshinskii
Caragana
19 alignments
Criteria: 70%, 100 bp
Regions: 257
Alignment 8
Oxytropis_diversifoliaÂ
Oxytropis
13 alignments
Criteria: 70%, 100 bp
Regions: 264
Alignment 9
Oxytropis_splendens
Oxytropis
11 alignments
Criteria: 70%, 100 bp
Regions: 264
100%
50%
100k
103k
106k
109k
112k
115k
118k
121k
124k

Galega_orientalis Galega_orientalis:1-125280

Alignment 1
Galega_officinalis
Galega
12 alignments
Criteria: 70%, 100 bp
Regions: 233

Alignment 2
Astragalus_mongholicus
Astragalus
13 alignments
Criteria: 70%, 100 bp
Regions: 264

Alignment 3
Astragalus_galactites
Astragalus
11 alignments
Criteria: 70%, 100 bp
Regions: 264

Alignment 4
Astragalus_laxmannii
Astragalus
15 alignments
Criteria: 70%, 100 bp
Regions: 264

Alignment 5
Calophaca_sinica
Calophaca
14 alignments
Criteria: 70%, 100 bp
Regions: 262

Alignment 6
Caragana_jubata
Caragana
15 alignments
Criteria: 70%, 100 bp
Regions: 267

Alignment 7
Caragana_korshinskii
Caragana
19 alignments
Criteria: 70%, 100 bp
Regions: 257

Alignment 8
Oxytropis_diversifoliaÂ
Oxytropis
13 alignments
Criteria: 70%, 100 bp
Regions: 264

Alignment 9
Oxytropis_splendens
Oxytropis
11 alignments
Criteria: 70%, 100 bp
Regions: 264

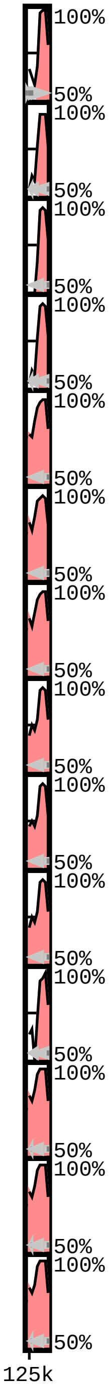

Supplement: Supplementary file 1 [file genes-14-00176-s001.zip › mVISTA of G. orientalis.pdf]
