# Supplementary figures and images for: Analysis of Complete Chloroplast Genome: Structure, Phylogenetic Relationships of Galega orientalis and Evolutionary Inference of Galegeae
Source: Genes (Basel). 2023 Jan 9;14(1):176. doi: 10.3390/genes14010176 (PMC9859028; doi:10.3390/genes14010176)

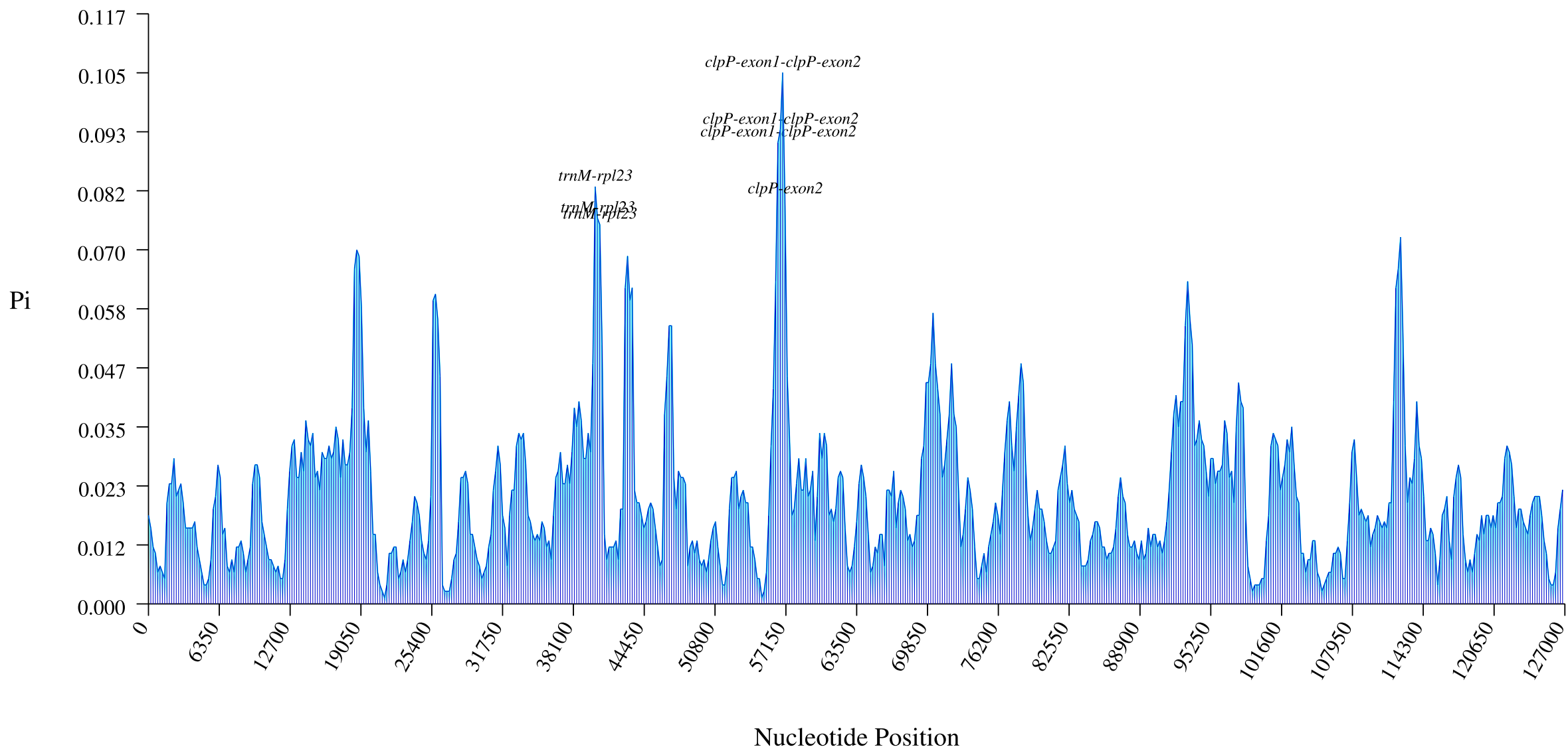

Supplement: Supplementary file 1 [file genes-14-00176-s001.zip › Figure S1 Pi of G.orientalis and G. officinalis.pdf]
